# Understanding Urban Green Space Usage through Systems Thinking: A Case Study in Thamesmead, London

**DOI:** 10.3390/su14052575

**Published:** 2022-02-23

**Authors:** Giuseppe Salvia, Irene Pluchinotta, Ioanna Tsoulou, Gemma Moore, Nici Zimmermann

**Affiliations:** Institute for Environmental Design and Engineering, The Bartlett Faculty of the Built Environment, University College London, London WC1H 0NN

**Keywords:** urban green space, system dynamics, systems thinking, unintended consequences, causal loop diagram, rapid ethnography, leisure time, social practices

## Abstract

Urban green spaces provide environmental, economic, societal and health benefits to cities. However, policy and planning interventions aiming to improve usage have often led to unintended consequences, including, in some circumstances, an actual decline in usage. Previous research has identified factors influencing the use of urban green space, more often with a focus on the ‘quality’ and physical features of the space, rather than on the broader social factors. This study aims to unpack the complexity of factors that influence the use of urban green space through the application of Systems Thinking. A qualitative mixed-method approach integrating System Dynamics with rapid ethnography was adopted to elicit the views of local residents in Thamesmead, London. A thematic analysis of interviews was undertaken to systematically map the causal relations between factors, which were compared to wider stakeholders’ views. Our findings highlight the relevance of dynamics and social influences on the use of green space, which include social interactions and stewardship, health conditions, availability of services and amenities. These are factors that are underexplored in the literature and, sometimes, overlooked in urban green space policy by decision-makers. We infer that attendance of urban green spaces requires time, which may be occupied in other practices determined by local conditions and needs. Expanding the spatial and temporal boundaries of investigation, wider than debates on ‘quality’, should, in our view, increase the chances of identifying critical influences and foster an increased use of green space.

## 1 Introduction

The importance of green space in urban areas (e.g., parks, woodlands, gardens, green-ways) is widely recognized by city officials and residents [1], as its multiple ecosystem services [2] provide environmental, economic, societal and health benefits to cities [3,4]. A large and diverse body of literature has studied the impacts of urban green space (UGS) on environmental sustainability and health that range from improved microclimate conditions (e.g., thermal comfort, air quality) [5–7] through increased value of nearby homes and land [8,9] and to enhanced social cohesion, recreation and physical activity [4,10].

Concerns over the preservation, creation and quality of urban green space are increasing in prevalence for a number of reasons: continuing trends of urbanisation, the intensification of urban areas, the move towards privatized spaces (i.e., gated communities) and the changing role of technology in connecting people [11,12]. Recent debates have been centred on the decline and potential revival of urban green spaces. Since the early 1990s, several countries across Europe and the United States have been implementing urban greening programmes, aiming to increase the quantity and quality of gardens and parks [13]. Nevertheless, recent studies have reported a decline in the use of UGS by citizens [14,15], including areas where such programmes operated [16]. Literature suggests a variety of influences for such decline, ranging from individual conditions (e.g., age, socio-economic status, health) to the physical features of spaces (e.g., greenery quality and quantity, facilities, proximity) [15,17,18]. Yet, although often studied in isolation, these influences are interlinked, producing a complex web of factors and determinants of use.

UGSs can be viewed as complex systems characterized by interactions and behaviours across multiple scales. Systems Thinking may capture how the multitude of influences interact on the use of UGS and explain undesirable effects of UGS programmes, e.g., [19]. This perspective suggests that micro-scale influences on the use of green areas and their effects in distant places and times may not be fully captured by decision-makers involved in urban greening strategies, including municipalities, urban planners and developers. The human capability to understand complex systems is limited [20] and decision-makers may neglect what eludes their selective attention [21] as well as their way of framing problems, e.g., [22]. Furthermore, such decision makers (e.g., planners and designers) may be focused on objective factors of the environment—including those reported by standard guidelines—which may be less influential than perceptions, for instance on people’s wellbeing [23].

Systems Thinking is a long-established interdisciplinary approach intended to holistically understand complexity, design policies and guide change, through the elicitation and improvement of ways of thinking, deciding and enacting by considering the interlinked influences of the system [24]. Besides capturing local knowledge, Systems Thinking and System Dynamics modelling approaches can help urban planning and implementation by building trust, consensus and long-term value [25–28]. Systems Thinking has been applied in an extensive range of areas. In urban planning, Systems Thinking and modelling studies inform on dynamics to be tackled for enabling the transition towards more sustainable and healthier cities [29–31]. Natural urban assets may contribute to this goal, as informed by some Systems Thinking studies. For instance, some studies map causal relations between socio-economic and political factors and their effects on the quality of the environment to support decision-making for the design, assessment and management of the natural space [22,32,33]. Some other studies adopt participatory Systems Thinking approaches to understand stakeholder views of the co-benefits from nature-based solutions and the implied trade-offs of benefits [34,35].

Marginally considered is the use of UGS by the local community in Systems Tthinking studies. Notably, through a participatory Systems Thinking approach, Zimmermann et al. [36] track stakeholder views on the benefits and unintended consequences of green infrastructure provision at the municipal scale; despite the use of such infrastructure results to play a crucial role in unleashing physical and mental well-being, the information on the determinants of use is limited to the perceived safety. On the other side of the spectrum, Svendsen et al. [37] focus on the dynamics of a specific type of use of green space, i.e., community gardens, as a proxy for improving health conditions in socioeconomically disadvantaged city neighbourhoods with concerns about asthma.

In summary, to our knowledge, what is missing is a detailed understanding of the causal relations in the use of common UGS, which could inform on the fundamental enablers for use increase. Our work investigates this research gap; specifically, it adds to a growing body of literature studying the use of UGS from the local community (i.e., residents and workers) perspective and employs a Systems Thinking approach as guidance to capture micro-level influences of usage. The aim of this study is to systemically map causal relations (i.e., factors) on the use of UGS in a London area according to the views of the local residents and workers. Our specific objectives are to explore:

1. the differences in professional views and local residents’ contextual knowledge to better understand the use of UGS and inform urban development, especially for healthier and more sustainable living;
2. the potential of participatory System Dynamics in visualizing the factors that influence use through causal maps.

In line with the recommendation of a recent literature review for a better understanding of the health impacts of green infrastructure [38], we intend to achieve these objectives with a qualitative system-based approach; this draws on ethnographic and participatory modelling for data collection in order to identify causal relationships in the use of UGS.

The article is structured as follows. The next section provides a brief literature review on the usage of UGS. Eventually, the case study is presented, followed by the methodology which describes data collection through rapid ethnography, as well as the construction and analysis of the causal map. The results section provides (a) general insights from the causal map analysis; (b) comparison with the views of organisational stakeholders; and (c) a close-up look at influential yet underexplored features. The discussion focuses on the relevance of social influences inside and outside of UGS. The study concludes with limitations and prospects for future work.

## 2 An Overview of the Literature on Urban Green Space Usage

In line with other framings, within this study, we define UGS as publicly accessible areas bearing some vegetation where use and interactions occur [39], mostly for recreational purposes (e.g., socializing, entertainment, relaxing and sports), such as urban public parks, gardens and children’s play areas [40]. The factors that influence the use of some UGS compared to others are wide ranging. Such factors might be physical barriers to access, such as roads, the quality or the condition of the open space itself and the size and the dimensions of the area. Some scholars suggest that it is necessary to focus on the morphology of UGS to urban green space planning [41,42]. The influencing factors may vary depending upon the perspective taken. For instance, when Coles and Bussey [43] used questionnaires and focus groups to examine attitudes towards woodlands amongst forestry professionals and local residents, they found distinct differences between professionals’ and users’ valuation criteria. Detailed ethnographies have illustrated that differences in the encounters with public spaces are no one thing—differences will always enter and be played out in UGS. For instance, Dines et al.’s [44] ethnographic study within East London demonstrated the social value of a range of informal open spaces related to the contribution the spaces made to peoples’ attachment with their local area. Similarly, in City Publics: The (Dis)Enchanments of Urban Encounters, Watson [45] explores the micro-publics of social contact and encounters in unusual sites and spaces. There is a need to be careful in these social interpretations; the meaning behind social values should be unpacked and differences acknowledged. For instance, age, gender and/or culture can result in dissimilar experiences—both positive and negative—of public spaces, as highlighted by Watson’s [45] notions of difference.

What is becoming clear is that to create quality spaces, it is crucial to design and manage spaces around how they will be used and to ensure that the spaces are relevant to community needs [43,46,47]. During the COVID-19 pandemic, parks have been a key resource and focus for health and wellbeing; this helps explain the substantial increase in usage of such spaces in a variety of cities—including London where this study operates—+160% according to [48]. There is a need for greater awareness and knowledge of the way in which the contexts of UGS are experienced and valued, or urban ‘space consciousness’ [44]. Exploring different stakeholders’ engagement with green spaces can help researchers understand these experiences, which can be used to improve design and promote usage. Decision-makers (e.g., local government, planners, designers, landscape architects) can ultimately benefit from knowing how the users experience the spaces, and what factors promote usage. Nevertheless, peoples’ relationships and connections with UGSs can be difficult to tap into, especially as the public’s language and conceptualisation of such spaces may be different to that of the designers and professionals who often created the spaces. The engagement of the local community in urban planning decisions can help in capturing diverse influences and local needs [49], thus increasing the chances of effective results, including increased use of UGS [16]. Incorporating local knowledge further contributes to ‘identifying gaps in expert assumptions, improving professional understanding of local practices, and highlighting culturally based health promoting practices’, although new ways of capturing knowledge are needed [50]; for this, participatory and co-design approaches to UGS design have produced positive results, e.g., [51].

## 3 The Case Study: Thamesmead, London, UK

Our study focuses on a UK-based case study in the London urban area of Thamesmead, where maintaining long-term quality of the natural—as well as built—environment and increasing its use is a priority in the redevelopment of the area [52].

**Figure 1:**
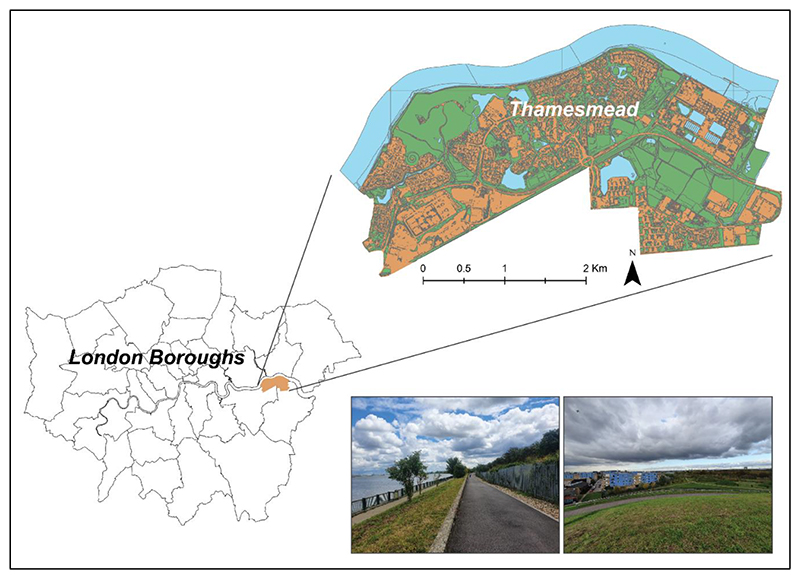
The area of the case study, Thamesmead. It is located in south-east London across two boroughs (**bottom-left**) and has an abundance of green space (green areas in **top-right** map, pictures of the area **bottom-right**). Base-map of London boroughs and green areas from London DataStore (https://data.london.gov.uk/dataset/statistical-gis-boundary-files-london (accessed on 9 July 2021) and https://data.london.gov.uk/dataset/green-and-blue-cover respectively (accessed on 9 July 2021)). Photos and graphical elaboration by the authors.

Thamesmead (Figure 1) is an urban area administered by two boroughs in south-east London. It hosts twice the amount of green space per person than the London average, as 150 of its 750 hectares are covered by parks, plus 14 sites of nature conservation interest and 32 hectares of water, consisting of five lakes (mostly embedded in green areas), canals and riverfront [53]. Nevertheless, UGS is reportedly underexploited by the local community [52].

Described as desolate [54], the Thamesmead architectural landscape is mostly constituted by concrete social-housing stocks built since the mid-1960s under an ambitious post-war housing project, which was never fully realised. The project produced a sheer amount of open space as perhaps its greatest legacy [55].

Nowadays, diverse vulnerabilities affect Thamesmead, including deprivation, unemployment and child poverty [56]. Reportedly, the area holds the record for the longest commute in the capital [57], which is possibly influenced by the area’s lower level of access to the city public transport network [58]. A one billion sterling regeneration plan—one of the biggest in the UK [59]—aims at improving the current conditions by leveraging on the natural capital as well [53]. The manager of the estate and initiator of the regeneration plan, Peabody Trust, commissioned the development of a framework to inform the regeneration; called ‘Living in the landscape’, this framework is grounded on five main programmes to harness the benefits of the natural environment to achieve the vision of a greener, more equitable and resilient Thamesmead in 2050 [60]. Additionally, an action research approach grounded on community participation is undertaken by a European Commission-funded consortium and project, called Clever Cities, to unleash the socio-economic benefits of implementing nature-based solutions in the area [61].

Our study forms part of a wider research project, which aims to conduct policy-relevant, actionable research to support cities to meet environmental imperatives, and to improve health and wellbeing [62]. Through working with stakeholders and decision-makers, this study aims to feed into the regeneration plan by anticipating potentially critical influences in the use of UGS through the integration of diverse local views with a Systems Thinking approach. The project involved a range of stakeholders in a multi-step participatory modelling process (Table 1, for details see [52]). After a set of preliminary scoping interviews mostly on perceptions of the problems affecting the case study, the stakeholders collaboratively defined a shared concern for the future of Thamesmead. A major social housing association, local authorities and environmental associations agreed to prioritize the long-term quality of natural and built infrastructures. Eventually, the influences on this concern were elicited within each stakeholder group, with the supplementary participation of local community and academic modellers.

In this paper, we focus on understanding how the natural and built infrastructures are integrated into everyday life in Thamesmead under the lens of one stakeholder group, i.e., the residents and representatives of voluntary and community sector local groups. In the following sections, we report on the building and analysis of causal loop diagrams (CLDs, stages three and four of the wider process, in Table 1), which visualize their views.

## 4 Methodology

This study is based on a qualitative approach, which is particularly useful to green infrastructure for understanding the richness of people’s lived experiences [38]. The methodology to build and analyse the resident-focused CLD integrates qualitative System Dynamics with rapid ethnography. First, qualitative System Dynamics included the construction of CLDs, a core concept of this discipline, which is intended to map feedback structures of systems [24]. CLDs mainly help to describe the complex set of interconnections and loops of influences in the use of UGS and to discover unintended consequences, including the reduction of use; to our knowledge, this study pioneers the development of a CLD for the use of green space.

Secondly, we used an ethnographic approach to elicit determinants and influences of the use of UGS. Rooted in anthropology, ethnography describes the life and structure of a community or social group from their own point of view [63,64]. Ethnography data are generally gathered from “what people say and do in certain situations in order to illuminate broader comparative questions” [65]. The use of this approach for theorising—rather than focusing on describing—is still debated among ethnographers [63]; our study leverages the capability to elicit and gather information for describing people’s life, which is eventually integrated with the System Dynamics approach to infer theories of UGS use. The research takes ethnographic approach both at a programme (e.g., CUSSH and CAMELLIA, see Funding section) and specific research project level (i.e., the case study of Thamesmead). At the programme level, we apply a multi-sited ethnographic approach, which operates in diverse locations, especially in order to understand ways for strategic collaborations between researchers and participants to emerge from the fieldwork [66,67]. This is suitable for addressing the transdisciplinary, multi-agency and multi-context international nature of the programme [68]. Within this particular case study, we are using rapid ethnography. Traditionally, ethnographic studies are carried out for a long time period in the environment of the community being studied; nevertheless, this may not always be feasible, as in our study. Rapid ethnography captures and triangulates social, cultural and behavioural information from multiple sources over a compressed period of time, which is important for “generating findings within time frames when they can still be actionable and used to inform improvements” [69].

Rapid ethnography allowed us to deliver relevant insights within tighter timelines and disruptions resulting from the COVID-19 pandemic. The pieces of information derived from both phone interviews and literature scoping were integrated in the building of the causal map (Section 4.2) for a more detailed view of the local community regarding the use of green space and were mapped with a System Dynamics approach to identify causal relations. This work complements the views and maps of the organisational stakeholders (Table 2), whose views were captured in three workshops intended for the collaborative, mostly synchronous production of a causal map (full details in [52]). The maps produced with the organisational stakeholders are compared with the one generated through residents in this article to identify the main elements of commonality and differences.

### 4.1 Interviews and Literature Scoping

Semi-structured interviews addressed the experiences of living in Thamesmead for the community, with the questions focusing on the elicitation of preferences of types of public places, activities undertaken in both domestic and public spaces and barriers to desired use of space; the interview guide is reported in Appendix A. The interviews were conducted in June and July 2020 with a purposive sample of seven people who lived or worked across different areas in Thamesmead.

Table 3 shows the local group in the voluntary and community sector represented by each interviewee; each interviewee is associated to a code, which is used in the following sections to identify the speaker of quotes from the interviews (e.g., INT1 for the first interviewee).

Four interviewees were identified by the researchers via an online search for local clubs, churches and social groups. One interviewee was known to the researchers as both a maintenance supervisor and resident who is passionate about the local natural environment. The remaining two interviewees expressed availability in a short questionnaire distributed by the researchers in particularly active Thamesmead-related Facebook social media groups. Despite being limited, the sample is comparable to the number of stakeholders who participated in the modelling workshops; furthermore, it elicited a considerable number of factors influencing the use of UGS.

The interviews were conducted over the phone or via a video-conferencing platform, and audio/video-recorded with the permission of the interviewee. Information about the interview’s objective and approach, data management and the overarching study were provided to the interviewee beforehand, as well as the possibility to respond to questions on these either before the interview or at the start.

Additionally, we consulted scientific and grey literature (i.e., reports) which addresses social aspects in Thamesmead and adjoining areas. Among the 36 pertinent publications, 10 included causal relations, which were embedded in the construction of the causal map, as described below and referenced in the results. The studies were undertaken or published between 1985 and 2019, with half of them in the concluding decade. This relatively extended timeframe covered by the publications allowed the possibility to identify if and how some elements of the area changed over time; nevertheless, some main elements around the use of green space described in recent studies echoed earlier ones.

### 4.2 Building the Causal Map

We undertook a thematic analysis of both literature and interview transcripts for the identification of influences and causal relationships to be represented in a CLD. CLDs are common tools in System Dynamics to represent in a schematic fashion (diagram) cause−effect interconnections between variables of a model, which generally form a closed path from a variable back to itself (loop). The interconnection or link between variables is positive if the variables change in the same direction (e.g., they both increase or decrease); a change in the opposite direction is represented with a negative link [24].

**Figure 2:**
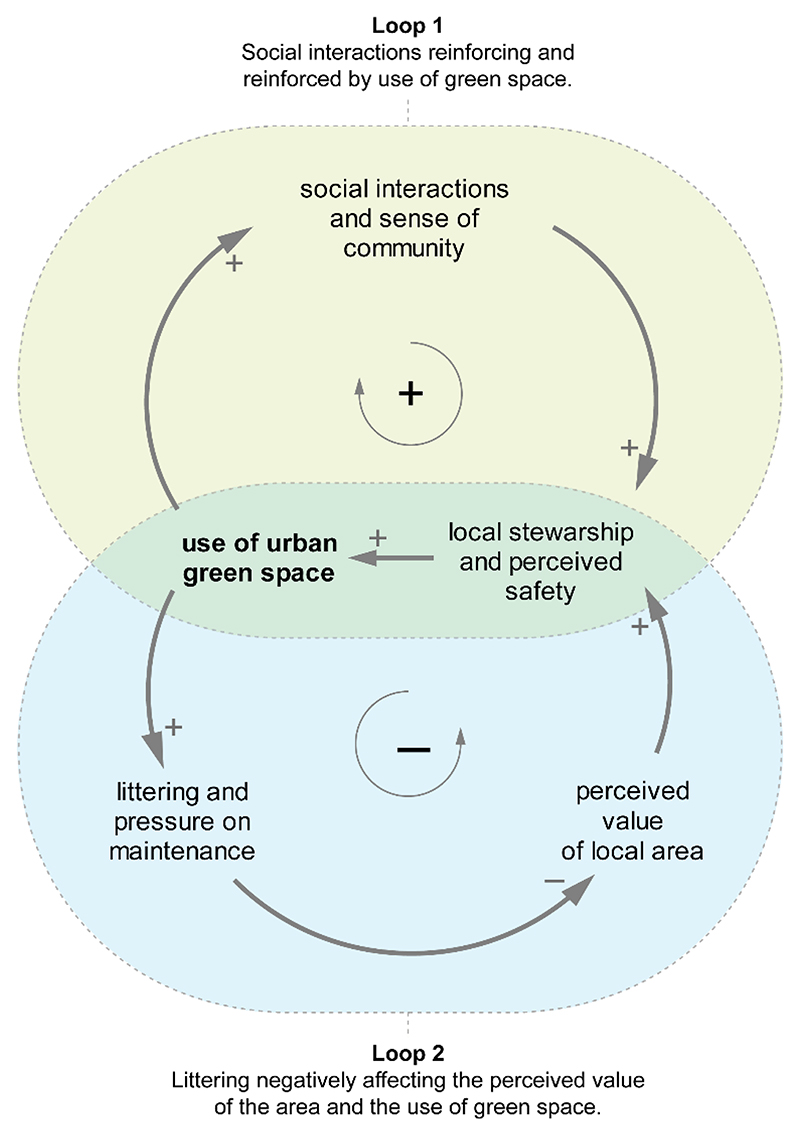
Main feedback loops in our study with opposite effect in response to changes in the use of UGS. In loop 1 (**top**), use of UGS increases with (and is increased by) an increase of social interactions, thus producing a reinforcing behaviour (sign + in the centre). In loop 2 (**bottom**), littering increases with an increase of use of green space; this negatively affects the perceived value of the area and as a consequence the use of UGS decreases, therefore producing a loop with a balancing behaviour (sign *−*).

Figure 2 shows a simplified CLD from this study, in which the use of UGS is affected by two feedback loops. The first loop embeds a positive link to and from social interactions, thus reinforcing the use of UGS; the second loop balances out the use by increased littering from increased use of UGS, which then reduced perceived value and use.

CLDs are important for identifying feedback mechanisms in the system, as humans often fail to include this in their understanding [24,70]. Within this paper, CLDs were used to describe the system in terms of causal connections and mutual influences, incorporating stakeholders’ views and ideas [35,71]. CLDs were also selected for their ability to map and visualize the network of interactions and influences among the different system components, making it understandable for non-experts and, therefore, facilitating discussion among stakeholders [28,72].

The methodology draws on former studies using purposive text analysis for the construction of CLDs [73,74] and is developed as follows:

1. Identifying variables (i.e., influences) and causal relationships in data, i.e., tracking sentences in which the variation of an element (e.g., increase or decrease) is explicitly associated with a change in another element.
2. Aggregating thematically similar variables under a common term, i.e., merging the elements identified in step 1 which indicate the same feature or topic.
3. Transforming text into words-and-arrow diagrams, which is a CLD, i.e., visualising the causal relationship between two elements (identified in step 1) by connecting their names (defined in step 2) through arrows.
4. Simplifying the CLD from redundant links, i.e., compressing longer chains of causal relationships (generated in step 3) when stemming was missing.
5. The causal relations reflect the understanding and perceptions portrayed in the sources, i.e., the interviews and publications.

### 4.3 An Analysis Method of the Causal Map

The CLD was analysed through quantitative and mostly qualitative methods, for the identification of (a) feedback loops and other features; (b) themes covered by the variables; and (c) the most linked variables.

The identification of the feedback loops as well as the building of the CLD was supported by the software Vensim (https://vensim.com/, accessed on 28 January 2022). The variables in the resident CLD were clustered into coherent themes generated by three of the authors, first individually and then collaboratively, until consensus was reached on the specific cluster to be associated to each variable of the CLD; this collaborative researchers’ approach to the cluster analysis intends to minimise the chance of biases that individual researchers may hold, and therefore it strengthens the robustness of the results.

Finally, we analysed the CLD to identify the most linked variables through the computation of the Degree Centrality (DC); this consists in the summation of direct in-arrows and out-arrows, and reflects the complexity of the network of links [75]. Changes in variables with high DC could generate a higher number of effects in the wider system represented by the map.

We compared the variable clusters and the DC for residents with those of the organisational stakeholder groups to identify possibly disattended influences (see [52] for additional information about the general analysis method and the specific results for organizational stakeholder CLDs). The outcomes of the resident CLD analysis along with the comparison with other stakeholder ones were presented and discussed with stakeholders, with the aim of informing and assessing the soundness of the approach and of the results.

## 5 Results

This section reports the following results of the analysis through the lens of the local community CLD and it is structured accordingly:

1. Constituents, i.e., variables and their links, feedback loops and clusters;
2. Commonalities and differences of the local community-focused CLD with the CLDs representing the views of the organisational stakeholder groups;
3. Focus on two interrelated loops.

### 5.1 Constituents: Variables, Feedback Loops and Clusters in the Resident CLD

The analysis of the literature and the interviews produced 74 and 52 recorded causal non-conflicting relations, respectively (steps 1 and 2 of the CLD building process). The records were reproduced into a CLD, composed of 69 variables and 103 links between them (steps 3 and 4). Despite the comparable number of variables, the number of links in the organisational stakeholder CLDs is about double, probably greatly influenced by the different method used for their production.

Approximately a third (*n* = 24) of the variables and three causal relations are identified both in the literature and interviews. The majority of the variables, including those with higher DC, are covered by only one of the two sources; this suggests that the interviews and the consulted literature are appreciably complementary (rather than duplicating each other) and contribute to identifying highly interrelated elements of the system.

The DC indicates that ‘perceived safety’ and ‘use of urban green space’ are the most linked variables, as well as being influenced with the top number of in-arrows; these are followed by ‘quality and numbers of local facilities (and things to do) for recreational activities’—the most influencing variable with the top number of out-arrows—and ‘social interaction opportunities’ (Table 4).

The ‘use of urban green space’ is included in almost every feedback loop (eight out of 10), followed in presence by ‘perceived safety’, ‘local stewardship’, ‘perceived value of Thamesmead’, and ‘littering and fly-tipping’ (column ‘No. of loops’ in Table 4); therefore, changes in these influences could likely reverberate in the wider system. Several loops overlap by sharing a consistent number of variables and links. Three are the main topics according to which the loops may be classified; these are described in Section 5.3 below.

**Figure 3:**
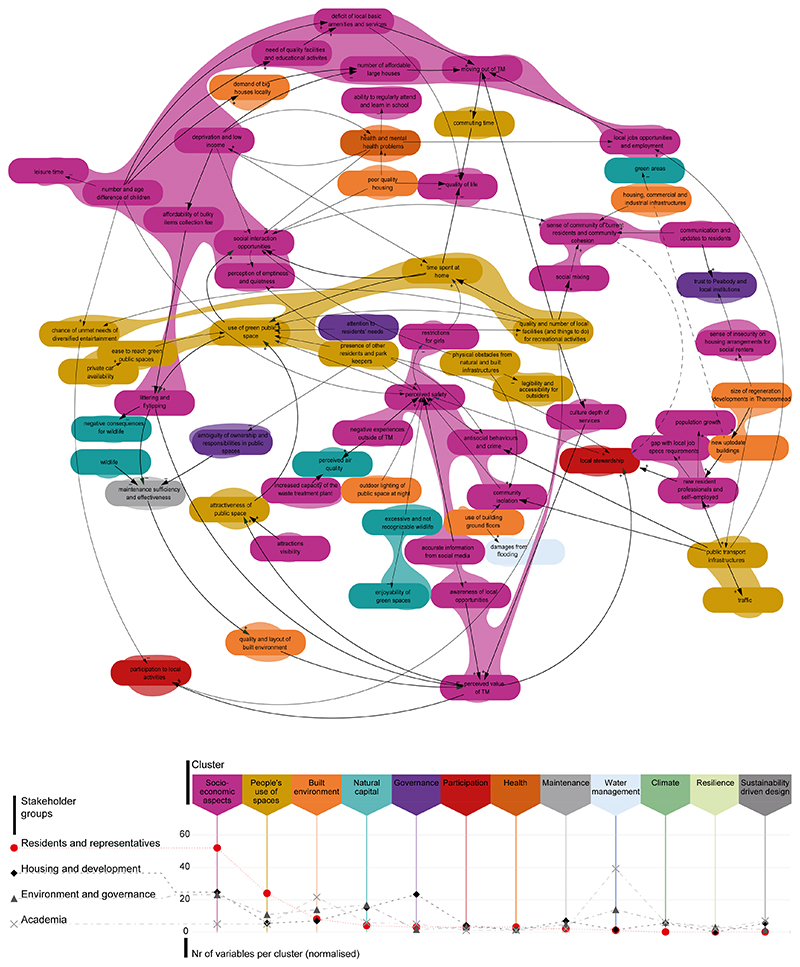
Analysis of the thematic clusters associated to the variables. These are visualized in CLDs (i.e., coloured areas, **top**) and in a comparative chart (conveniently connected marks counting the normalised number of variables per thematic cluster, **bottom**), specifically for residents and across stakeholder groups.

The resident CLD covers about half (*n* = 21) of the thematic clusters identified across all the stakeholder groups, with up to five clusters in one single loop; such abundance emphasizes the complexity of elements involved in the use of UGS (Figure 3). The socioeconomic aspects cluster (pink areas in Figure 3) dominates with about half of the variables associated to this (*n* = 32), followed by ‘people’s use of spaces’ (*n* = 13). These two clusters are also the most represented per DC.

### 5.2 Comparison with Organisational Stakeholder Groups

The relative dominance of variables associated to social aspects and the use of space is distinctive of the local community CLD, whereas the organisational stakeholders’ ones show a substantially different representativeness of clusters (Figure 3, bottom). Namely, the socio-economic aspect is a top cluster for the housing association and of the environment and governance groups, yet with an inferior relative presence. The second top clusters per number of variables for these groups are governance and natural capital, respectively, which are instead absent or nearly so in the resident CLD.

Drastically different is the cluster ranking for the academia CLD, dominated by water management and followed by the built environment; both clusters are substantially less represented or absent in the resident CLD.

Generally, key aspects of the resident CLD describing social interactions and the phenomenon of spending time outside the area are missing in the CLDs of the organizational stakeholders. Conversely, some relevant features for these stakeholder groups are missing in the resident CLD; these include biodiversity, natural capital performance, climate change, water quality, investment programme and funding mechanisms.

Joint foci for local community with some other groups include affordable housing (environment and governance group); stewardship (environment and governance group plus housing association); time restrictions, low income, and deprivation (academia plus environment and governance group); safety related issues and how their perception may be influenced by design features (housing association). Despite being the most central variable of the local community CLD, perceived safety is missing or less relevant in the system understanding of the other groups.

Additional variables linked directly or indirectly to the use of natural space across the groups include accessibility and ease to reach it, information and awareness of the local opportunities and of the value of the local area.

Likewise, stewardship (high DC for local community) is linked to the use of green space in different ways by other stakeholder groups. It is framed as affected by the use of natural spaces for the environment group, and by the implementation of co-design approaches for the housing and development group. In either case, stewardship is not integrated in any loop including perceived safety, as it is for the local community CLD.

Finally, parenting duties are reflected in only one variable across other groups (‘children’s education’ for the housing association), which negatively affects the availability of leisure time, as per the local community’s view.

### 5.3 Variables and Loops Influencing the Use of UGS

Residents and community representatives identified that the use of UGS in Thamesmead is directly affected by a multitude of variables. The interviews and literature revealed that the usage of such spaces was expected to increase with an increase of: its visibility, thus attracting more transient visitors (INT5, i.e., Interviewee 5); availability of private cars and therefore accessibility [47]; and the offer of recreational facilities and initiatives which better fit residents’ needs [47,56]. Improvements in the provision of green space and things to do may increase not only opportunities for social mixing (INT3) and the perceived value of the area for the community (INT1), but it more likely will also meet the needs of larger, multigenerational families [56]. Ultimately, recreational opportunities within the local green space may reduce time spent at home (INT3; [56]) or outside of Thamesmead (INT5), possibly in favour of using the local UGS.

Our analysis identified two loops of particular significance in influencing green space usage, which are: (1) littering and perceived quality of the environment; and (2) social interactions and time spent outdoors locally; this time is also influenced by a number of practices outside Thamesmead. These loops and practices are examined in the following sub-sections.

#### 5.3.1 Feedback between Safety and Littering

**Figure 4:**
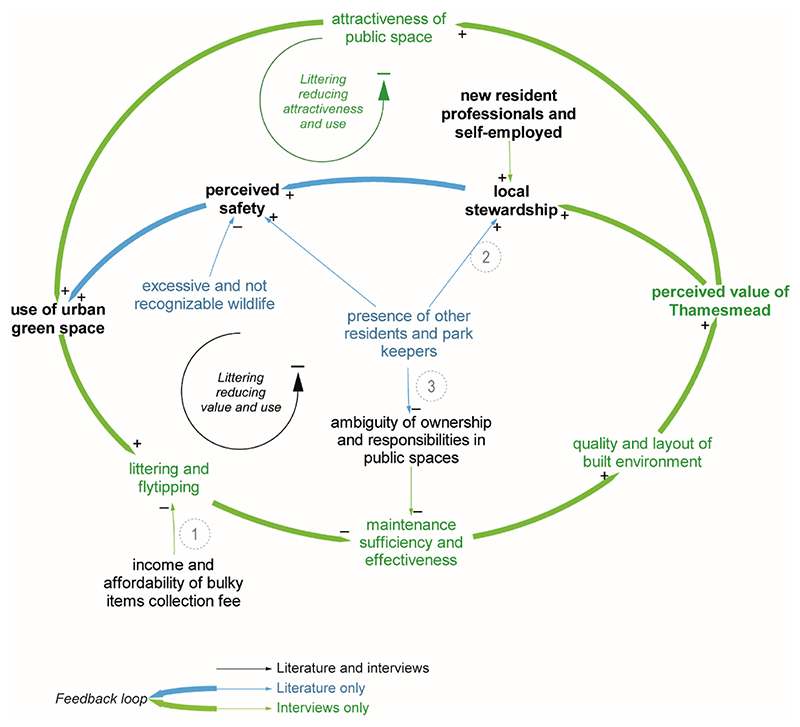
Close-up on loop engaging littering, which negatively affects pressure on maintenance, perceived value, ultimately the use of UGS.

As depicted in Figure 4, perceived safety affects the use of UGS, especially for parents [56]. Its perception is shaped by several factors (high DC), and it relates to residents’ former experiences in other city areas (INT2), the spatial environment and social norms. Environmental factors with a negative effect on perceived safety include excessive and unrecognized wildlife [47], as well as estate arrangements and infrastructural provision, such as poor lighting, scarce services activity—especially in the ground floor—and lack of night-time economy [55]. These conditions may increase the perception of being in an unsafe UGS or area to pass through.

Social norms around safety are affected by anti-social behaviours and crime, which discourage residents from walking around specific areas, especially at night [56]. Nevertheless, recent literature [47,56] as well as an interviewee (INT1) agree about the frequently inaccurate news regarding local (or even non-local) crime; this may foster scaremongering especially on social media, with consequences on perceived safety.

Perceived safety links to two main feedback loops involving either social interactions or littering (Figure 2); these two reciprocally balance their reciprocal (de-)growth [56] and therefore the effects on the use of UGS. This suggests that the higher use of UGS comes at a cost, particularly for the maintenance service, because the increased littering may discourage further use.

The littering loop (Figure 4) results from the analysis of the interviewees’ anecdotes, which point to two possibly relevant causes: the unaffordability of the waste collection fee for some households (INT1; link 1 in Figure 4) and the perceived negative effects of littering on higher park attendance, for example, during the COVID-19 pandemic (INT5).

Few variables in this loop are influenced by the ‘presence of other residents and park keepers’, including children and male friends. Park keepers and gardeners foster stewardship behaviours (link 2) and safety (link 3) with positive effects on the use of UGS; they remove the ambiguity of ownership in open spaces with their symbolic role (link 4), as they embody ‘the collective right to access to open space without fear of abuse or attack’ [47].

#### 5.3.2 The Loop about Social Interactions and Time Spent at Home

**Figure 5:**
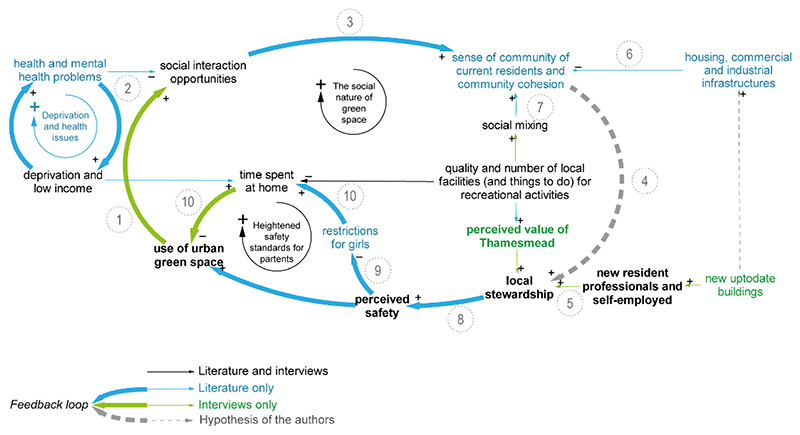
Close-up on loops on socials interactions and cohesion, in which perceived safety affects the time spent at home and therefore the use of green space. Links reported in the text are numbered in the figure.

Figure 5 shows the reinforcing nature of social interactions in the use of UGS, an area less explored in greenspace literature. Events and initiatives taking place in UGS are believed to increase opportunities for social interactions outdoors (INT3, link 1). According to the literature, social interactions in public space are affected by urban infrastructure layout [76], health issues [56] and deprivation [77]; the latter two are linked in a vicious cycle which may cause social exclusion (links 2).

Historic reviews of the social and spatial polices of Thamesmead note the mismatch between planners’ aspirations and communities’ social practices. The desire for a precise and ordered spatial form, in the housing regeneration in the 1980’s and 1990’s, went against traditional (working-class) practices (of public life as socializing, mixing, noisy, accidental and spontaneous) and thus the redesign possibly unconsciously reinforced a distrust of this community life [78]. We hypothesize that a sense of community increases stewardship and careful resident behaviours with respect to their local public spaces (link 4); we consider this highly plausible both in general and with respect to the experiences and perceptions reported by the interviewees.

Regeneration plans may produce contrasting effects on both local stewardship and sense of community. According to an interviewee, the regeneration could attract professionals working in a nearby business district; these new residents are expected to appreciate the area and to exhibit more careful behaviour than established residents (INT6, link 5). Nevertheless, literature suggests that past developments in Thamesmead have reduced the sense of community and its cohesion (link 6, [47]); some perceive detachment and limited social mixing, especially between middle-class residents living in new developments and working-class residents in surrounding neighbourhoods (link 7, [79]). Social mixing is anecdotally reported to be uncommon across the three main communities immigrating to Thamesmead in different periods.

In a past study on the use of UGS in Thamesmead, the collapse of communal care and therefore of local stewardship is associated with a reduction of perceived safety (link 8, [47]). Three decades later, safety continues to represent a major concern; parents limit children’s UGS attendance, especially that of girls (link 9), both nowadays for African families [56] and in the mid-1980s in general [47]. In such conditions, more time is spent by families at home or in their immediate surroundings rather than local UGSs (INT2, links 10).

#### 5.3.3 Practices Outside Thamesmead

**Figure 6:**
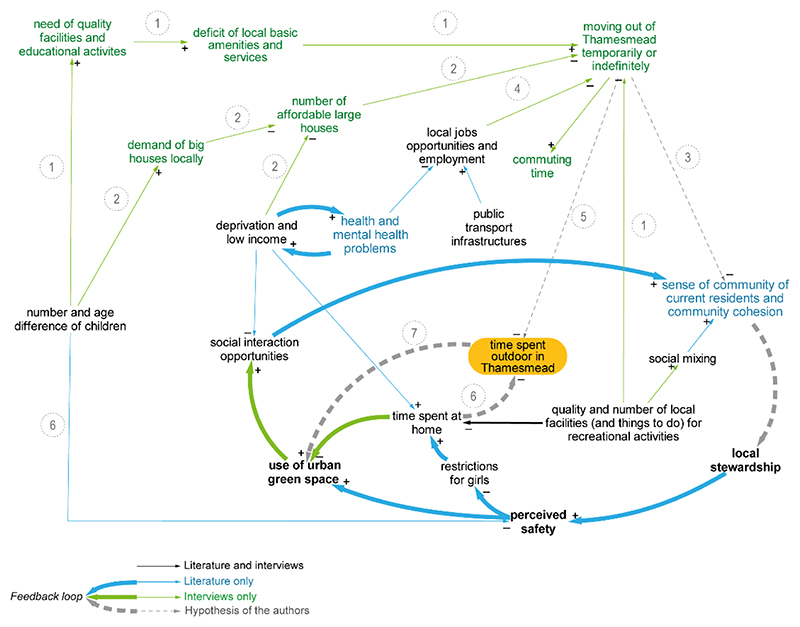
Hypothesis of parenting duties and scarcity of local facilities limiting the time spent outdoor in Thamesmead and therefore in local UGS.

Literature and interviews report on local life and several practices, i.e., routinized types of behaviours interconnecting artefacts, skills and meanings [80,81]. A number of described practices and issues in Thamesmead points to a particular outbound trend; this is represented in Figure 6 by the variable with a high DC, ‘moving out of Thamesmead temporarily or indefinitely’. These two timeframes are integrated to represent a relevant phenomenon for the aim of this study, i.e., the distancing of the community from the local UGSs.

Mostly raised by one interviewee (INT3), this phenomenon is absent both in the literature and in the CLD created by the other stakeholders; hence, we wish to illuminate this connection. While the other groups involved in the research recognise the effects of time constraints, they seem to miss the link to amenities, which they may have the power to influence within their professional remits. Nevertheless, the consequences on the use of UGS may be substantial. This outbound move is generally associated with larger families, seeking educational and entertainment activities which meet their needs. The quality and variety of local facilities and amenities, including parks, is reported as insufficient, especially for families with teenagers, and secondary schools are almost absent (INT3, links 1). In such conditions, families generally struggle to participate in local activities, ‘as there isn’t always something to keep all children across the age groups occupied and entertained’ [56].

‘People go out of Thamesmead [… for] clothes shopping. […] You got a little shop here, but you know people want other stuff. Then you’ve got people going off Thamesmead for restaurants and pubs […]. The things we have got in Thamesmead is a KFC and a Mc Donald’s. If you wanna go to a restaurant you have to go a couple of miles to get to it.’

(INT5)

Furthermore, families are said to relocate to other areas with affordable larger houses, which are reportedly scarce in Thamesmead, as many have limited financial resources (INT3, links 2). Permanent relocation weakens previously established relationships, with negative consequences on the sense of community and social cohesion, and therefore on the use of space (link 3).

In summary, some of the vulnerabilities which characterize the local area—e.g., larger family needs, infrastructural deficit, high rates of deprivation and unemployment (link 4)—are associated by the residents to an outbound movement; this outbound movement ultimately reduces the use of UGS in Thamesmead by reducing the sense of community or by reducing the time available to be spent outdoors (link 5). If increased time is spent either at home (links 6) or outside of Thamesmead, it can be inferred that less time is available to be spent in the local UGSs (link 7).

## 6 Discussion

Our case study has contributed insights into understanding the interconnections between factors that influence the use of UGS. Some features and topics emerging from our investigation resonate with past research; for instance, the clusters identified in our analysis resonate with most of the themes used by a review about the value of urban green space in England [82]. Nevertheless, causal relations between these themes are less explored. Our findings, drawing on the perceptions of the local community, suggest ‘notions of difference’ [45] in factors that influence usages between different stakeholders. We have gained an understanding of the use of System Dynamics to reveal the connections between different factors. In doing so, our research has highlighted the value in taking a Systems Thinking approach to conceptualising and understanding UGS usage. In this section, we consider our findings in relation to existing research and practice. First, we describe the limitations of our approach. Then, we discuss our insights, considering how our findings connect with previously reported factors that influence green space use.

With regard to limitations, we recognize that data collection and validation was deeply affected by the disruptions of the COVID-19 pandemic. Future work should aim at a higher number of interviews, to be complemented by observations in green areas for more detailed accounts of practices and influences. The multiple sources used for the production of the CLD provided a richer yet complex understanding at the expense of consistency (e.g., terminology, spatial and temporal frames, methods). Ideally, the structure and the effects emerging from the causal map would have been discussed with the interviewees collaboratively; however, mandatory social distancing prevented in-person activities (e.g., focus groups or workshops), whereas virtual environments can hinder productive conversations with people less familiar with them. Despite these limitations, through a Systems Thinking and rapid ethnography approach, we integrated two main sources of information (i.e., interviews and literature) for the construction of a causal map. The credibility of our study is reinforced by the alignment of findings within the wider literature.

### 6.1 The Social Nature of Green Space Use

The resulting CLD from this study outlines the diverse factors related to the usage of UGS. The quality and abundance of facilities available is influential, which is shown from the high DC as well as being consistent with a multitude of studies, e.g., [83,84]; nevertheless, they constitute only part of the wider system. A set of influences on the use of UGS in Thamesmead reported three decades before [47] remains persistent, according to our interviewees and the more recent literature; these include accessibility, perceived safety and ambiguity of ownership. Whereas some factors identified in the CLD are widely explored in the literature (e.g., safety, accessibility, built environment), other determinants of the quality and use of UGS, such as social cohesion and interactions among the local community, are overlooked by organisational stakeholders.

Although social cohesion has a top DC value in our study, this is underexplored according to literature reviews on human–environment interactions in UGSs [3,4]. The exposure to UGS has long been reported to foster social integration and cohesion, e.g., in a USA-based study [85]. Delving into the social nature of UGS use and the influence of social cohesion is extremely relevant in contexts characterized by limited social mixing, especially if estate developments may further segregate, as our sources suggest. In fact, visitors of green areas may feel no need to interact with anyone other than their own social group [86]. For instance, particularly busy green areas may disincentivise some people, especially those with a greater interest in the natural features rather than the social interactions, as reported by a study in Brussels [87]; this effect may be related to the concern over infections during the COVID-19 pandemic, which may weaken the motivating role of engaging in social interactions in green space [88]. Furthermore, our study pointed out that the quality and quantity of facilities for recreational activity affects the level of social mixing and therefore the sense of community. The quality of green space is associated to opportunities of social interactions and cohesion for some studies, namely, those conducted in Hong Kong [89] or in Turkey [90] in relation to a sense of neighbourhood attachment. This causal link is unsolved in the literature, as emphasised by Yu [89]. His study demonstrates the relevance of quality of the environment, rather than quantity of facilities; nevertheless, this result is acknowledged to be inconsistent with other similar studies, thus deserving further investigation.

Likewise, the organisational stakeholder CLDs lack the ambivalent effects of newcomers attracted by the regeneration plan on local stewardship, social mixing and perceived safety. Some studies emphasize the need to explore the variety of uses and meanings associated with social groups, and ethnic minorities in particular, for supporting their use of UGSs [3,91,92]. Therefore, consideration is needed of such social and cultural determinants if the use of UGS is to be fostered, because modifications of the built environment alone—a cluster with high DC for several stakeholder groups—may be insufficient to trigger significant changes of behaviours, namely healthier active practices [e.g., 84].

In line with insights from other studies [3,93] and earlier works of urban scholars [94], our cluster analysis and the DC ranking confirm the dominant socio-economic nature of the influences of UGS for residents. This cluster is remarkably less represented or almost absent from the organisational stakeholder CLDs.

Possibly, the high representation of both socio-economic aspects and use of space derives from the scope of the ethnographic investigation, which was intended to capture life patterns and experiences in Thamesmead. Alternatively, the dominant clusters within each group CLD seem to resonate with the sector represented by the stakeholders. Bounded in an environment of decision, organisational structures and routines [20,21], the attentional capability of decision-makers may fail to acknowledge important socio-economic influences in the use of UGS.

Longer temporal frames and larger spatial scales of reference in procedures and plans may lead organizations to disattend local granular features and priorities [95], which are instead typical of residents’ accounts of daily life and routines. Therefore, pairing and comparing the views of both local community and organisational stakeholders is recommended to reduce the chances of neglecting relevant aspects at different scales, inside and outside the UGSs, as discussed below.

### 6.2 Green Space as the Place Where Time-Demanding Practices Occur

Our analysis of social influences emphasizes how the use of UGS relates not only to their built environment-related features, but also to those characterizing the population and the wider area. Notably, the top DC ranked variables include deprivation, health problems, number of children, time spent at home and moving out of Thamesmead temporarily or indefinitely. Especially the latter two suggest the importance of understanding what people do or do not do outside UGSs in order to foster their use. UGS is used for accomplishing practices, typically walking, playing with children and eating or drinking outdoors according to interviewees and [96]; Lee et al. [84] stress that the activities—rather than the space—are beneficial, especially to health. Nevertheless, almost absent in the literature is the understanding of ‘the many ways in which visits to, activities in and experiences of urban greenspace are integrated in the everyday lives and life courses of urban populations’, considering the possibilities and flexibility that these spaces provide [97]. Increasing the use of UGS requires an understanding of the needs which are or may be met there. Our interviewees reported a multitude of activities and routinised practices that carry residents outside of the local area (e.g., shopping, entertainment, educational activities), in addition to social factors keeping them at home. According to a 10-year survey about English citizen engagement in the environment [96], being ‘too busy at work’ and ‘too busy at home’ are the most common reasons for not spending more time out of doors.

Daily practices occupy and compete for time and performers [81]. In other words, increased time spent by more people in UGS implies that activities occurring in other urban areas will be either deprived of time (e.g., less commuting, shopping) or performed in green spaces, perhaps simultaneously with other practices (e.g., doing exercise, meeting other people, entertaining children or relaxing, as was shown in our study). Either case requires spatial and temporal rearrangements of the constellation of practices occupying time at home or outside UGS, in order to include—possibly additional—activities in habitual patterns. Routines and ways of doing must be reshaped. Furthermore, emergent practices require repeated performance over time to persist [81]; therefore, fragmented interventions (e.g., occasional events) may not necessarily trigger the desired routinized patterns of UGS attendance. Studies including reflections about time tend to focus on the duration of activities undertaken in the use of UGS, e.g., [96,98]. To our knowledge, neglected is the exploration of the sequencing and rhythms which lead or do not lead to the use of UGS, with exceptions focused on social segregation [99,100]; in addition, previous studies have not included the multiple and dynamic interaction of a variety of influences.

We infer and recommend that the boundaries of investigation and interventions concerning the use of UGS be expanded to understand what time constraints and practices occur outside of UGS, spatially as well as temporally; this is reflected by the introduction of the variable ‘time spent outdoors in Thamesmead’ and the links to this in the CLD (Figure 6).

## 7 Conclusions

This paper developed and analysed a causal map to identify influences in the use of UGS in Thamesmead for the purpose of informing more effective environmental management as well transitions towards sustainable and healthy cities. The CLD reflects the views of the local community and complements work undertaken to understand the views of professional stakeholders with an interest in the redevelopment of Thamesmead. The focus on capturing contextual knowledge through the integration of different sources of information generated a multiplicity of benefits. First, this study contributed to knowledge of the influences on the use of UGS; most notably, these include the underexplored social elements of community interactions and cohesion, perceptions of the environment and local norms, concomitant or competitive needs and routines. These insights were gained through an ethnographic approach which elicited the knowledge of the local community to complement the views of other stakeholders. Second, the improved description of the case study presented in this paper informs organizational decision makers about factors and interconnections in the UGS space which may be distant in space and time, as visualised by the CLD, thanks to our Systems Thinking approach. Finally, we expanded the conventional boundaries of investigation to capture the complexity of UGS use. UGS cannot be seen in isolation—as a confined bubble within the city—but rather as part of a wider interconnected urban system in which practices occur. Focusing on the quality of UGS solely as a factor that influences usage may be insufficient to capture the complexity of the elements involved in its use or non-use. In this view, the set of influences and influential activities to be investigated for a better understanding of UGS use should be broadened, both spatially and temporally, in order to include practices that occupy residents in other places, either indoors or in other urban areas, at various times.

Drawing on our findings, leverage points for increasing UGS use may lie in different and apparently distant parts of the urban system; here, we propose possible strategies. The increase of local job opportunities and income could likewise increase the affordability of local services and activities as well as leisure time gained from reduced commuting. Similarly, maximising investment in public transport and active travel modes could increase accessibility and proximity for a wider population by reducing commuting time to reach UGSs as well as other services. Encouraging and establishing additional local services and business could equally reduce the temporary movement of residents outside the area and therefore spare more time to be spent locally and in UGSs. Finally, providing more support to the numerous larger families that struggle to keep children across age groups entertained with the limited infrastructure and free services available could enable their participation in the community.

Prospects for future developments include the identification of the most time-demanding practices for those who use UGSs less often, especially across diverse socio-economic groups.

## Supplementary Material

Appendix

## Figures and Tables

**Table 1:** Multi-step participatory qualitative modelling process informing the study, including the specific objectives, undertaken activities and resulting outcomes at each stage. With respect to the wider case study presented in [52], this article addresses stages 3 and 4 (background colour in grey) regarding the Causal-Loop Diagram building and analysis, respectively, especially about local community views of the use of urban green space, to be compared with organisational stakeholders.

Stage and Objective	Activity	Outcome
1. Problem scoping	Interviews with stakeholders	List of individual concerns about the case study
2. Shared concern definition	Workshop with organisational stakeholders	Agreed focus of investigation
3. Causal-Loop Diagram building	Series of workshops with organisational stakeholders	Four Causal-Loop Diagrams, one per stakeholder group
	Interviews with local community	
	Literature scoping	
4. Causal-Loop Diagram analysis	Cluster analysis	Recurrence of clusters
	Centrality Index calculation	List of most connected variables
	Structure analysis	Main features and loops
5. Prioritisation	Workshop with organisational stakeholders	Definition of focus for the subsequent quantitative modelling phase.

**Table 2:** Stakeholder groups involved in the whole research process; these include residents and their community representatives as well as organizational stakeholders in the sectors of housing and development, environment and governance, and academia. The organizational stakeholders operate in the area of this study and were identified through snowballing.

Stakeholder Group	Composition Description
residents and representatives	local residents and representatives of local groups for sport, social inclusion, religion and the environment.
housing and development	employees of multiple departments within the social housing estate owner and manager.
environment and governance	employees of multiple organizations, including local government, water utility company, environmental NGO, and environment agency.
academia	university research in hydrological modelling.

**Table 3:** List of interviewees of the study, including their area of action (if representatives of local voluntary and community sector groups) and code, for quotes reported in the text.

Represented Community Sector (and Role)	Interviewee Code
sport association (president)	INT1
religious community (reverend)	INT2
children support project (founder)	INT3
religious community (vicar)	INT4
natural environment stewardship team (supervisor)	INT5
resident	INT6
resident	INT7

**Table 4:** Top 20% variables per DC in the resident CLD, number of feedback loops which involved them and the thematic cluster associated to them.

Variable (Top 20%)	DC	No. of Loops	Cluster
perceived safety	11	7	Socio-economic aspects
quality and numbers of local facilities (and things to do) for recreational activities	8	0	People’s use of spaces
social interaction opportunities	8	3	Socio-economic aspects
use of urban green space	8	8	People’s use of spaces
perceived value of Thamesmead	7	6	Socio-economic aspects
deprivation and low income	6	1	Socio-economic aspects
health and mental health problems	6	1	Health
public transport infrastructures	6	0	People’s use of spaces
time spent at home	6	4	People’s use of spaces
littering and fly-tipping	5	6	Socio-economic aspects
moving out of Thamesmead temporarily or indefinitely	5	0	Socio-economic aspects
new resident professionals and self-employed	5	0	Socio-economic aspects
number and age difference of children	5	0	Socio-economic aspects
presence of other residents and park keepers	5	0	People’s use of spaces

## Data Availability

Data collected and generated by this research is considered confidential.
