# Supplementary material for: Understanding Urban Green Space Usage through Systems Thinking: A Case Study in Thamesmead, London
Source: Sustainability. Author manuscript; Available in PMC 2024 Oct 21. (PMC7616722; doi:10.3390/su14052575)
Supplement: Appendix [file EMS199296-supplement-Appendix.pdf]

Appendix A

Guide of the semi-structured interviews with residents and members of the local community.

Date	[yyyymmdd]
Interviewee code	[TMR *]
Interviewer	[initials]
Accepted informed consent	[Yes, option *)]
Interviewee	[Name]

Questions	Prompt
A. GREETINGS, INTRODUCTION AND INFORMATION SHEET	
Hi, this is [Name] from [University]; I am approaching for the interview on Thamesmead that we arranged few days ago.<br>Is it ok for you to proceed now?<br>The interview will take about 20 min.	If not available ask to reschedule already.
Thanks for accepting this short interview which will be very useful for the research project on life in London, including Thamesmead.<br>Did you have chance to read through the document called information sheet that I sent you?<br>[If Yes] Do you have any questions about this?<br>[If No] No problems at all. I will read it to you, if you cannot read it by yourself now, and please feel free to raise any questions or doubts.<br>As soon as this is done, I will ask to confirm that recording the conversation is ok for you and, if you wish, a different name may be used for yourself during the conversation. For sure data will be anonymised in any publication which may include some reflections from the interviews.	
Recording	
Activate recording	
Information sheet	
Read aloud while recording	
B. YOUR LIFE IN THAMESMEAD (TM)	
1. For how long have you been living or working in TM?	
2. How do you spend your day in TM, before the lock-down, of course?	"Think of an average day and your routines"<br>Which is the area and the places in TM more familiar to you?
Top things in TM	
3. What are the things and the places in TM that you like and appreciate the most?	Naming landmarks and street names first, then also Built, Blue and Green spaces (BBG), e.g., parks, gardens, lakes, canals, playgrounds, library, community centre...<br>Online. Show a map too (not necessarily easy to navigate though)<br>"For instance, possibility to chill out and relax, chance to meet other people"
4. Can you say that these places improve your quality of life in Thamesmead? And are there other places in TM for which you say "this place fits with my life and priorities, they improve the quality of my routines and life here"?	
5. Do you think that most people in TM would share the same experience of yours? Consider the people who you engage with the most	
(If not included in the above response)<br>5.b. Could you describe a bit more the people you had in mind for the last question? Are they people who you have the chance to meet and engage with?	Where do they live<br>age range<br>Profession<br>Parenting duties
C. BARRIERS	
6. Are there things and activities that you would like to do or do more but you cannot for any reason?	If not reported spontaneously, ask "what does prevent you to enjoy them"?
D. Built, Blue and Green environment<br>(if time allows)	

Questions	Prompt
7. You mentioned [place name/perception] Please could you elaborate a bit more on that?	
What about instead [another place]?	
Long term quality	
8. What do you think may keep or improve the quality of (IN TURNS, slowly) green spaces, water spaces and buildings in TM for the next years to come? In other words, what will make your life better in TM?	Specifically,<br>green spaces, like parks<br>Spaces with water like rivers and canals<br>Buildings and constructed places
D. CONCLUSION<br>Additional contacts	
Thanks for your answers. We are reaching the end.<br>9. Before closing the interview, I would like to ask if you have in mind someone who may consider to talk with us as you have just done?	
Thanks and closing	
Thanks for your time and precious support. Please feel free share any comment or questions now or in future to contact me if you require additional information, clarifications or any other reason.<br>If you do not mind, we might contact you back for future activities.	
Stop recording	
Thanks and goodbye!	
